# A Role for *Caenorhabditis elegans* COMPASS in Germline Chromatin Organization

**DOI:** 10.3390/cells9092049

**Published:** 2020-09-08

**Authors:** Marion Herbette, Valérie Robert, Aymeric Bailly, Loïc Gely, Robert Feil, David Llères, Francesca Palladino

**Affiliations:** 1 Laboratory of Biology and Modeling of the Cell (LBMC), CNRS, Ecole Normale Supérieure de Lyon, Université de Lyon, 69007 Lyon, France; marion.herbette@ens-lyon.fr (M.H.); valerie.robert@ens-lyon.fr (V.R.); loic.gely@ens-lyon.fr (L.G.); 2 Centre de Recherche en Biologie cellulaire de Montpellier, CRBM, CNRS, University of Montpellier, 34090 Montpellier, France; aymeric.bailly@crbm.cnrs.fr; 3 Institute of Molecular Genetics of Montpellier (IGMM), CNRS, University of Montpellier, 34090 Montpellier, France; robert.feil@igmm.cnrs.fr (R.F.); david.lleres@igmm.cnrs.fr (D.L.)

**Keywords:** COMPASS, SET1, condensin, chromatin, FLIM-FRET, *C. elegans*, germline, pachytene

## Abstract

Deposition of histone H3 lysine 4 (H3K4) methylation at promoters is catalyzed by the SET1/COMPASS complex and is associated with context-dependent effects on gene expression and local changes in chromatin organization. The role of SET1/COMPASS in shaping chromosome architecture has not been investigated. Here we used *Caenorhabditis elegans* to address this question through a live imaging approach and genetic analysis. Using quantitative FRET (Förster resonance energy transfer)-based fluorescence lifetime imaging microscopy (FLIM) on germ cells expressing histones eGFP-H2B and mCherry-H2B, we find that SET1/COMPASS influences meiotic chromosome organization, with marked effects on the close proximity between nucleosomes. We further show that inactivation of *set-2*, encoding the *C. elegans* SET1 homologue, or CFP-1, encoding the chromatin targeting subunit of COMPASS, enhances germline chromosome organization defects and sterility of condensin-II depleted animals. *set-2* loss also aggravates germline defects resulting from conditional inactivation of topoisomerase II, another structural component of chromosomes. Expression profiling of *set-2* mutant germlines revealed only minor transcriptional changes, suggesting that the observed effects are at least partly independent of transcription. Altogether, our results are consistent with a role for SET1/COMPASS in shaping meiotic chromosomes in *C. elegans*, together with the non-histone proteins condensin-II and topoisomerase. Given the high degree of conservation, our findings expand the range of functions attributed to COMPASS and suggest a broader role in genome organization in different species.

## 1. Background

The spatial configuration of chromatin is essential to ensure fundamental processes from gene expression to cell divisions [1], but how higher-order structures are formed in various cellular processes remains unclear. Mitosis and meiosis are essential cellular functions that require restructuring and reorganization of chromatin architecture, and are both associated with specific changes in histone modifications. During mitosis, extensive compaction of chromatin is associated with histone H3 serine 10 phosphorylation (H3S10ph), H4 lysine-20 mono-methylation (H4K20me1), and a reduction in overall histone acetylation [2]. Specific histone PTMs, including H3 lysine-4 tri-methylation (H3K4me3), are also associated with meiotic double strand breaks (DSBs) during recombination, and are dynamically altered during meiotic progression [3,4]. In addition, during mammalian spermatogenesis a large fraction of the observed dynamic changes in H3K4me3 do not coincide with either gene promoters, or double strand breaks (DSBs) [5], suggesting additional functions.

SET1 family histone methyltransferases act in large multi-subunit complexes known as COMPASS (complex proteins associated with Set1) to deposit H3K4me3 at promoters of actively transcribed genes [6,7]. At promoters, levels of COMPASS-dependent H3K4 methylation generally correlate with mRNA levels, but evidence for an instructive role for H3K4me3 in transcription is lacking, and recent data suggest that its function depends on different chromatin and cellular contexts [8].

Studies of individual components of COMPASS are consistent with a role in various aspects of chromatin organization. For example, in yeast binding of Spp1/CFP1 to H3K4 is required to tether loop formation and DNA cleavage at sites of double strand breaks (DSBs) [3,9,10,11], while Set1 plays H3K4-dependent and -independent roles in genome organization through long-range clustering of retrotransposon loci [12,13]. In developing mouse oocytes, inactivation of CFP1 results in defects in meiotic oocyte maturation, spindle assembly and chromosome alignment, with only minor effects on transcription [14].

In *Caenorhabditis elegans,* inactivation of *set-2*, encoding the single SET1 homolog, results in defective patterns of H3K4me3 in the germline, increased genome instability, and loss of germ cell identity leading to sterility [15,16,17,18]. Whether these defects reflect a direct role of SET-2 in transcription, or a more general role in germline chromatin organization is not known. We found no correlation between COMPASS-dependent H3K4me3 and transcription in *C. elegans* embryos [19], which is consistent with observations in other organisms and argues against a direct role in transcription [20,21,22,23]. Likewise, increased genome instability in *set-2* mutant germlines was not associated with defects in the transcriptional induction of the DNA damage response (DDR), suggesting downstream defects in the DNA repair process [15]. Altogether, these results evoke the possibility that SET-2 can modify chromatin structure, at least in part independently of specific changes in gene expression.

In this study, we used fluorescence lifetime imaging microscopy (FLIM) for Förster resonance energy transfer (FRET) measurements to directly assess changes in chromatin compaction in live animals lacking COMPASS components. We find that FRET between fluorophore-tagged nucleosomes is significantly decreased in meiotic cells from *set-2* and *cfp-1* mutant animals, supporting a role for COMPASS in influencing close nucleosome proximity. Consistent with a role for COMPASS in chromosome organization, we found that loss of either *set-2* or *cfp-1* enhanced chromatin compaction defects in germ cells depleted of condensin-II, a major regulator of chromosome structure [24]. *set-2* inactivation also enhanced germline phenotypes associated with conditional alleles of condensin-II subunit *hcp-6* and *top-2*, encoding the chromosome structural protein topoisomerase-II [25,26,27]. Altogether, our data suggest that COMPASS contributes to germline chromosome architecture, and that COMPASS-related complexes may more generally contribute to higher-order chromatin structure together with non-histone architectural proteins.

## 2. Results

### 2.1. Nanoscale Chromatin Compaction Is Decreased in Set-2 Mutant Germlines

In the *C. elegans* germline, meiotic nuclei are arranged in a temporal-spatial order, with the distal end of the gonad containing mitotically proliferating nuclei. Homolog pairing initiates downstream in the “transition zone”, followed by the pachytene stage, during which synapsed chromosomes appear in DAPI-stained nuclei as discrete, parallel tracks. More proximally, nuclei exit pachytene, enter diplotene, and cellularized oocytes containing condensed homologs are formed [28].

H3K4me3 is detected on chromatin in all germline nuclei, from the distal mitotic region through the meiotic stages and into diakinesis (Figure S1A, [16,18]. In germlines from animals carrying the *set-2(bn129)* loss-of-function allele [18], H3K4me3 strongly decreases in the distal mitotic region through early-mid pachytene. Levels of H3K4me3 are not visibly altered in late pachytene and diakinetic nuclei of mutant animals, most likely reflecting the additional activity of SET-16/MLL, the only other SET1 family member in *C. elegans* (Figure S1A, [16,18,29]. *set-2(bn129)* animals at the permissive temperature (20 °C) have a reduced brood size, while at the stressful temperature of 25 °C a progressive loss of fertility is observed, resulting in sterility at the F6-F8 generation [16,18].

DAPI staining of chromatin revealed no apparent defects in either germline organization, or chromosome morphology in *set-2* mutant animals at 20 °C, or late generation fertile animals at 25 °C (data not shown and [18]. However, in late generation (F4) germline nuclei from animals approaching sterility at 25 °C, meiotic progression and chromatin compaction were altered, with a loss of the distinctive pachytene nuclei morphology (Figure S1B). The stressful temperature therefore reveals a role for *set-2* in germline function and chromosome organization.

To investigate how COMPASS influences chromatin architecture specifically in germline cells, we used a recently developed FLIM-FRET technique to quantify changes in chromatin compaction in live animals at the nucleosomal level. The assay is based on the measurement of FRET interactions between fluorescently-labelled core histone GFP-H2B (donor) and mCherry-H2B (acceptor) [30]. Transgenic animals expressing the two H2B fusion proteins did not show any obvious cell division, growth or reproductive defects [31]. Important features of this system include the following [31]: (1) H2B fusion proteins only represent approximately 4% of total histone H2B, so that only a minute fraction of total nucleosomes is expected to contain both tagged H2B histones as a potential source of intra-nucleosomal FRET; (2) GFP and mCherry are fused to the N-terminus of histone H2B, and the distance separating them from the histone proteins is too large (130 Å on average) to produce significant intra-nucleosomal FRET; (3) FRET occurs efficiently only when the donor and acceptor fluorescent fusion proteins are closely positioned (<10 nm) in the 3D nuclear space following chromatin compaction. Importantly, FLIM-FRET also provides accurate quantification due to the independence of the fluorescence lifetime from the relative concentrations of the interacting proteins, and is independent of their diffusion rates, ruling out any perturbation resulting from the kinetics of core H2B histones [32,33]. In summary, our assay measures close contacts in the nuclear space between distant nucleosomes, and thus provides a read-out of nanoscale chromatin compaction [34,35,36,37].

**Figure 1:**
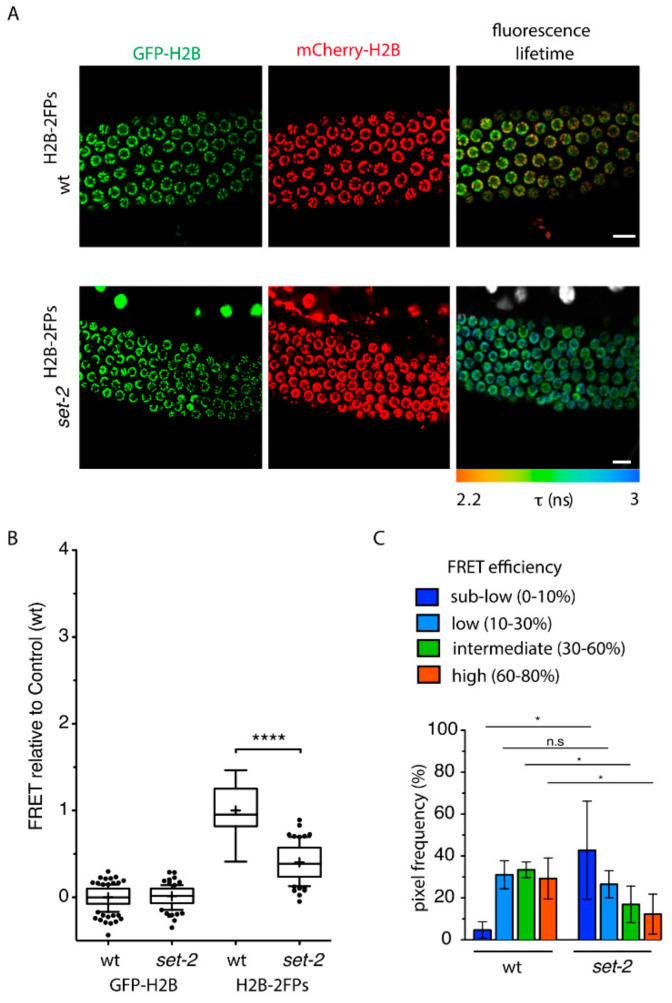
*set-2* inactivation influences nanoscale chromatin compaction in the germline. (**A**) Representative confocal images of pachytene-stage germ cells expressing GFP-H2B (green) and mCherry-H2B (red) fluorescent proteins from wildtype (wt ^H2B−2FPs^) (top) or *set-2(bn129)* ^H2B−2FPs^ mutant animals. The corresponding fluorescence lifetime images revealed the spatial distribution of the mean fluorescence lifetime (τ) at each pixel of the region of interest. Fluorescence lifetime values are represented using a continuous pseudo-color scale. Scale bars, 10 μm. (**B**) Graph box-and-whisker plots of the mean FRET efficiency relative to control (wt) showing that *set-2* nuclei show reduced chromosome compaction. The mean FRET value is indicated by a cross in each box. **** *p* < 0.0001 (two-tailed unpaired *t* test). (**C**) Histogram plots showing the relative fraction of FRET populations (sub-low, low, intermediate, and high) from wt and *set-2(bn129)* pachytene nuclei. The FRET populations represent the different levels of compaction as defined previously [31]. * *p* < 0.05; (two-tailed unpaired *t* test); n.s, non-significant. n = 5 gonads (approx. 350 nuclei) for wt, n = 6 gonads (approx. 430 nuclei) for *set-2(bn129).*

To carry out the FLIM-FRET assay, we first generated wildtype and *set-2* mutant strains that stably co-express both GFP-H2B and mCherry-H2B fusion proteins (FPs, collectively named “H2B-2FPs” hereafter) from a single transcription unit driven by the germline-specific *pmex-5* promoter (Figure 1A). We confirmed that there was no alteration in the expression of fluorophore-tagged H2B histones in *set-2* mutants (Figure S2A), and fluorescence recovery after photo-bleaching (FRAP) showed that the tagged histones H2B were homogeneously incorporated into chromatin (Figure S2B).

Comparative FLIM-FRET analysis of *set-2(bn129)*^H2B−2FPs^ and wt^H2B−2FPs^ pachytene nuclei revealed a strong reduction in chromatin compaction levels in the absence of *set-2*, as indicated by a longer GFP-H2B fluorescence lifetime (Figure 1A), and a reduced mean-FRET efficiency (Figure 1B). From the measurement and spatial mapping of FRET in individual nuclei of wt^H2B−2FPs^ pachytene-stage cells, we observed discrete regions associated with distinct FRET efficiencies across individual nuclei (Figure 1A). As previously described [31], based on FRET quantification we arbitrarily defined several classes of FRET, from “sub-low FRET” to “high-FRET”, that correspond to different levels of nanoscale compaction. Although there was some degree of heterogeneity between individual nuclei, we observed that in the absence of *set-2* “intermediate-FRET” and “high-FRET” populations previously linked to heterochromatic states [31] were overall significantly reduced compared to wild type (Figure 1C), while the “sub-low-FRET” chromatin class associated with more accessible chromatin was increased. These results suggest that the absence of *set-2* results in changes in nanoscale chromatin structure in the germline, and more specifically disrupts highly compacted states.

### 2.2. Loss of Set-2 Enhances Defects in Germline Chromatin Organization Resulting from Condensin-II Knock-Down

Defects in chromatin organization can result in chromosome segregation defects in mitosis and meiosis [38], but these were absent from *set-2* mutant germlines and embryos at all of the temperatures tested [15,19]. However, we previously showed that at the permissive temperature (20 °C), endoreplicated intestinal cells of adult animals show chromosome segregation defects [19] that are very similar to those reported in condensin-II mutants [39]. This suggests that in *set-2* mutants, subtle defects in chromatin organization may arise that become apparent only when chromatin structure is further perturbed.

**Figure 2:**
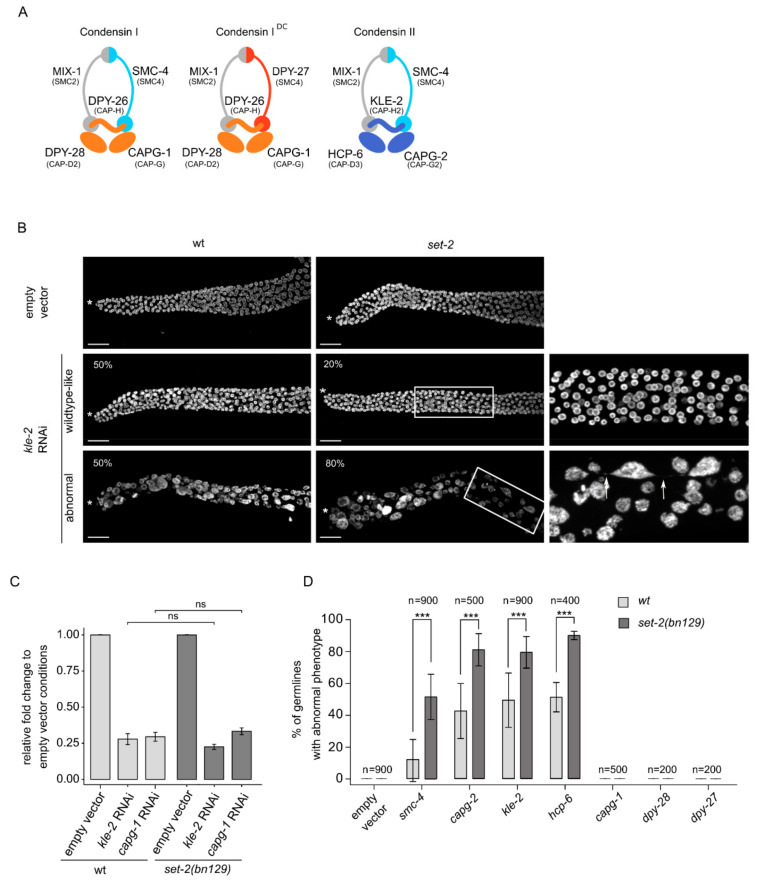
*set-2* inactivation enhances condensin-II depletion phenotypes. (**A**) *C. elegans* condensin subunits and their vertebrate homologs. (**B**) Confocal images of DAPI-stained distal germline region from wildtype and *set-2(bn129)* animals treated with empty vector or *kle-2* RNAi. (*) marks distal end of the gonad. Representative images show examples of “wildtype-like” and “abnormal” phenotypes, with their presence indicated as percentage (%) of total (n = 900, from 9 independent biological replicates) (scale bar, 20 μm). Arrow indicates the presence of chromatin bridge (scale bar, 10 μm). Images correspond to a Max intensity projection using using Fiji. (**C**) *kle-2* and *capg-1* mRNA levels in wildtype and *set-2(bn129)* mutant animals after RNAi directed against the respective genes. Relative fold change was calculated with respect to empty vector condition, following normalization with *pmp-3* and *cdc-42*. [*] *p* < 0.05 *t*-test. (**D**) Percentage of germlines with “abnormal” phenotype after RNAi directed against condensin-II (*smc-4*, *capg-2*, *kle-2* and *hcp-6*), condensins I (*capg-1*, *dpy-28*) and condensin I^DC^ (*dpy-27*) in wildtype or *set-2(bn129)* mutants. n = number of animals scored from 9 independent experiments for *smc-4* and *kle-2*, 4 for *hcp-6*, and 5 for *capg-1* and *capg-2*, and 2 for *dpy-28* and *dpy-27*. All scoring was performed in blind. *** *p* < 0.001 (*t*-test).

Condensins are major contributors to chromosome structure and organization [24]. Metazoans contain two types of condensin complexes (I and II) that share a heterodimer of two SMC (structural maintenance of chromosomes) proteins, SMC2 and SMC4, and are distinguished by three unique CAP (chromosome-associated polypeptide) proteins named CAPD, CAPG and CAPH [40]. Uniquely, *C. elegans* has an additional complex, condensin-I^DC^, which contributes exclusively to dosage compensation in somatic cells [41]. KLE-2, HCP-6 and CAPG-2 are condensin-II specific subunits, CAPG-1, DPY-26 and DPY-28 are common to the two condensin-I complexes, whereas DPY-27 is specific to condensin-I^DC^ (Figure 2A) [39]. In *C. elegans*, condensin-II associates with sister chromatids in meiosis and mediates their compaction and resolution [42,43].

Because knock-out of condensin-II subunits results in sterility, we could not study the interaction of single condensin-II mutants with *set-2*. Instead, we knocked-down different subunits in wildtype and *set-2* mutants by growing animals from the L1 larval stage to adulthood on condensin RNAi feeding plates, followed by scoring of DAPI stained germlines by fluorescence microscopy (Figure 2B,D). We initially focused on *kle-2* and *capg-1* RNAi to knock-down condensin-II and condensin-I complexes, respectively. RT-qPCR analysis showed that RNAi treatments resulted in a similar decrease in transcript levels in both wildtype and *set-2* mutant animals (Figure 2C), confirming that the efficiency of RNAi was the same in both genetic contexts.

Condensin-II RNAi resulted in reduced fertility in both wildtype and mutant animals. Because many of these animals also showed egg-laying defects, we were unable to use the number of viable progeny as a read-out of germ cell production and quality in wildtype animals compared to mutants. We instead used visual scoring of the germlines, placing animals in one of two broad classes: “wildtype-like” or “abnormal” (Figure 2B,D). Germlines in the wildtype-like class consisted of nuclei undergoing all stages of meiotic progression as in wildtype, although the total number of germ cells was reduced, consistent with the severe under-proliferation observed in condensin-II mutants [39]. The second class defined as “abnormal” consisted of severely disorganized germlines containing fewer and larger nuclei, often showing more intense DAPI staining (Figure 2B,D). Using a lacO/lacI-GFP system composed of a stably integrated lacO array and a lacI::GFP fusion protein able to bind LacO repeats [44], we observed multiple spots in enlarged nuclei, revealing that these were aneuploid nuclei (Figure S3). The abnormal germline morphology of these mutants made it difficult to clearly distinguish different region of the germline, and individual cells could not be unequivocally assigned to a specific meiotic stage. Nuclei were sometimes connected by thin chromatin bridges (Figure 2B, arrow), consistent with the known involvement of condensin-II in chromosome segregation in the germline and soma [39,45].

RNAi knock-down of the condensin-II subunit *kle-2* in wildtype animals resulted in a comparable number of germlines falling in the wildtype-like and abnormal class (Figure 2B). *kle-2*(RNAi) in *set-2* mutant animals resulted in similar phenotypes, but there were significantly more germlines showing an abnormal phenotype, representing 80% of all germlines in blind scoring experiments. Similar results were observed following RNAi knock-down of the other condensin-II specific subunits, *hcp-6* and *capg-2,* and of *smc-4*, common to both condensin I and II complexes. In all conditions, phenotypes were consistently and significantly more severe in *set-2* mutant compared to wildtype animals (Figure 2D). Knock-down of the condensin-I specific subunits *capg-1* and *dpy-28*, or the condensin-I^DC^ subunit *dpy-27* [39,42,43], did not produce any apparent germline phenotype, either alone, or in the *set-2* mutant background (Figure 2D). The effectiveness of *capg-1*, *dpy-28* and *dpy-27* RNAi was confirmed by scoring the associated dumpy (Dpy) phenotype in wildtype and mutant animals (Figure S4; www.wormbase.com).

In summary, germline phenotypes resulting from condensin-II knock-down are significantly and reproducibly more severe in the absence of *set-2*, suggesting that *set-2* may act with condensin to shape meiotic chromosomes.

### 2.3. Loss of Set-2 Differentially Affects Germline and Somatic Phenotypes of the Condensin-II Mutant Hcp-6(mr17)

**Figure 3:**
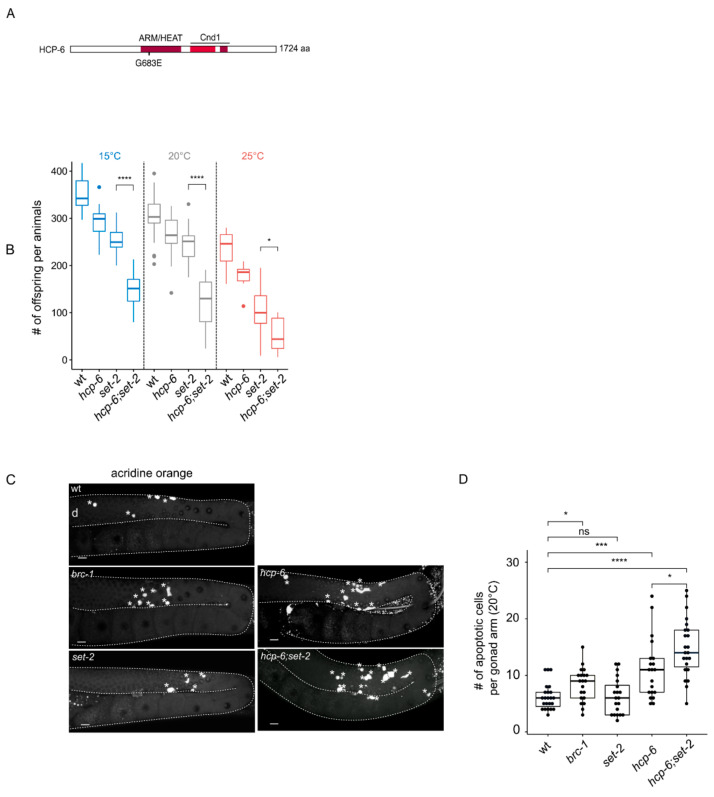
Enhancement of *hcp-6(mr17)* phenotypes in *set-2(bn129)* mutant animals. (**A**) Schematic diagram of HCP-6 protein and position of the *mr17* mutation. Conserved ARM/HEAT and Cnd1 (Condensin complex subunit 1) domains are highlighted in red. (**B**) Brood size per animals at indicated temperatures (15 °C, 20 °C, 25 °C), (n = 11 animals per genotypes at 15 °C and 25 °C, and 33 at 20 °C). [***] *p* < 0.001, [*] *p* < 0.05 (*t*-test adjusted for multiple comparison with the Bonferroni method). (**C**) Representative confocal images of germlines stained by acridine orange. Asterisks indicate apoptotic cells, (d) indicates distal region; orientation is the same for all gonads. (**D**) quantification of the number of apoptotic cells in the germline of animals switched at 20 °C for 24 h (n > 20 gonads per genotypes). A Wilcoxon test was performed after a significant difference with a Kruskal Wallis test, [ns] non significative difference, * *p* < 0.05, *** *p* < 0.001, **** *p* < 0.0001.

As an alternative to the RNAi knock-down approach above, we used *mr17*, a hypomorphic allele of the condensin-II subunit *hcp-6* that carries a missense mutation resulting in temperature-sensitive embryonic lethality due to chromosome segregation defects [45]. In their previous analysis, the authors identified this allele as a G to A transition at nucleotide position 3073 within the coding region, converting glycine 1024 to glutamic acid [45]. We could not confirm the presence of this mutation by resequencing. Instead, we identified a glycine to glutamic acid substitution at amino acid 683 within the HEAT repeat region of HCP-6 in 3 independent experiments (Figure 3A). The reasons for this discrepancy are not clear, but may reflect errors in sequencing in previous experiments.

Because the germlines of *hcp-6(mr17)* single and double mutants are severely disorganized at 25 °C (Figure S5A), we were unable to visually score distinct phenotypic classes in single compared to *hcp-6(mr17);set-2* double mutants, as we did for the RNAi experiments. However, we observed that *hcp-6(mr17)* mutants showed a significant reduction in the number of progeny laid at all temperatures tested (15, 20 and 25 °C) (Figure 3B), consistent with the essential role of condensin-II in germline chromatin organization and fertility [39]. As previously described [18], *set-2* single mutants showed reduced fertility. Brood size was further and significantly reduced in *hcp-6(mr17);set-2* double mutants compared to either of the single mutants at all temperatures (Figure 3B). The *hcp-6(mr17);set-2* double mutant phenotype was most severe at the non-permissive temperature of 25 °C: double mutants laid a mean of 30 embryos per animal, compared to 250–300 for wildtype. Therefore, germ cell viability is severely impacted in these animals, most likely reflecting germline chromosome segregation defects [39].

Defective germline nuclei in the *C*. *elegans* germline are eliminated by apoptosis [46]. Using the dye acridine orange to mark apoptotic cells [47], we observed as expected an increase in apoptosis in *brc-1/*BRCA1 mutants [47], while *set-2* single mutants were not affected (Figure 3C,D, [15]. The number of apoptotic corpses increased in *hcp-6* mutants, with a significant further increase in *set-2*;*hcp-6(mr17)* double mutants. Apoptosis may therefore actively eliminate germline nuclei containing chromosomal abnormalities, thereby contributing to the reduced fertility of these animals (Figure 3C,D). Alternative, or in addition, a decrease in the mitotic stem cell population may affect this phenotype. Altogether, these results further support a functional interaction between condensin-II and *set-2* in the germline.

The *hcp-6(mr17)* allele also allowed us to explore whether *set-2* genetically interacts with condensin-II in the soma. L4 animals raised at the permissive temperature of 15 °C were shifted to 20 °C and allowed to develop into adults, and their progeny scored. Under these conditions, the *hcp-6(mr17)* mutation resulted in greater than 85% embryonic lethality that was significantly reduced to 65% in *set-2*;*hcp-6(mr17)* double mutants (Figure S5B). The effect of *set-2* was not due to the suppression of cell division defects in these embryos*,* because *set-2*;*hcp-6(mr17)* surviving animals developed into adults showing phenotypes commonly associated with such defects, including uncoordinated behavior (unc) and sterility [48]. Together, these results suggest that *set-2* and condensin-II subunit *hcp-6* have distinct functional relationships in the soma and germline.

### 2.4. Functional Links between SET-2 and Chromosome Structural Protein TOP-2 in Germline Organization

Topoisomerase II is another major component of mitotic chromosome. When temperature-sensitive topoisomerase II mutants are used to bypass its essential requirement in mitosis, defects in chromosome condensation and segregation are observed in meiosis [49,50,51]. To explore whether *set-2* also interacts with *top-2* to ensure proper chromosome condensation in the germline, we constructed *set-2;top-2* double mutants using a recently described allele, *top-2(it7)*, that results in a temperature-sensitive chromosome segregation defects in male spermatogenesis [52].

**Figure 4:**
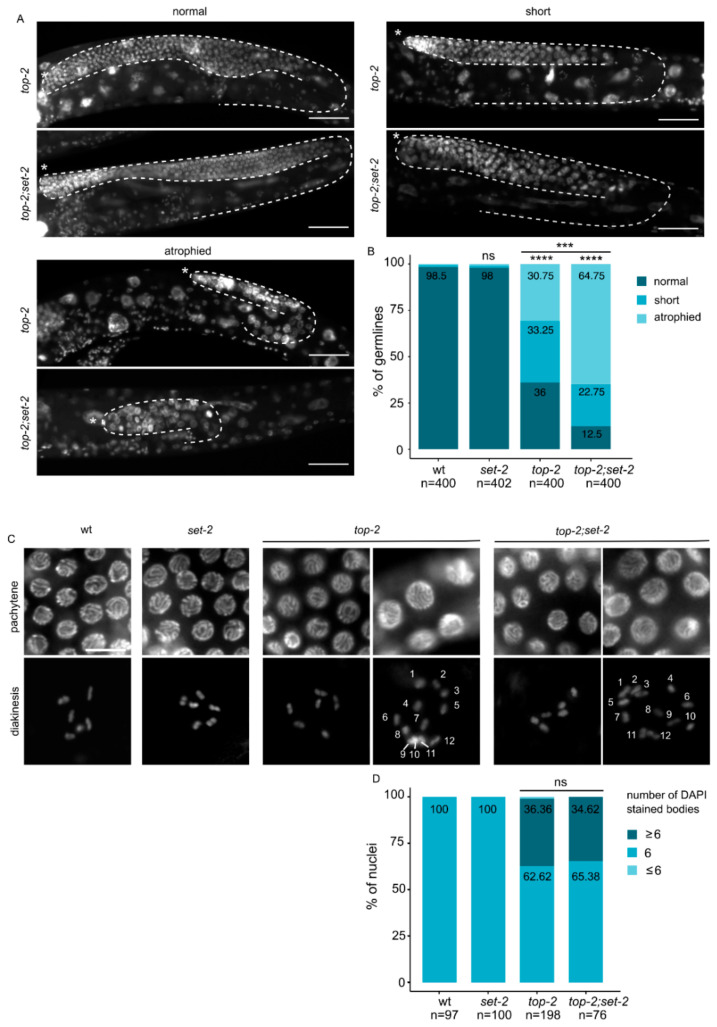
Enhancement of the *top-2* mutant phenotype in absence of *set-2***.** (**A**) Representative images of DAPI stained adult germlines showing different phenotypic classes. (*) marks distal end of the gonad (**B**) Scoring of phenotypic classes. Animals were shifted to 24 °C at the L1 stage and allowed to develop to adulthood. Germlines categories are as defined in Materials and Methods. **** *p* < 0.0001, *** *p* < 0.001 significant difference between mutant backgrounds using chi-square test and FDR correction (ns: non-significant). Scale bar, 50 μm. (**C**) Enlargement of pachytene and diakinetic nuclei from wildtype or mutant animals. For *top-2* single and *top-2;set-2* double mutants, representative nuclei from normal (left) and short (right) germlines are shown. Nuclei containing more than 6 DAPI stained bodies were observed in both “short” and “normal” germlines. (**D**) Scoring of aneuploid nuclei from cells in diakinesis (Scale bar, 10 μm).

*top-2(it7)* hermaphrodites that developed from animals shifted to the non-permissive temperature (24 °C) at the L1 larval stage had various defects in germline organization. 36% of adults contained normal germline arms whose size and developmental transitions were comparable to wildtype (Figure 4A). The remaining animals had either a smaller germline, such that germline bends were premature, or a germline atrophy phenotype with only a small population of mitotic germ cells. Animals that displayed germline atrophy were mostly devoid of germ cells at various stages of meiosis [27], and nuclei were larger in size, consistent with defects in chromatin compaction. Short and atrophied germlines with decompacted nuclei were significantly more abundant in *top-2(it7);set-2* double than in *top-2* single mutants, accounting for 65% of all germlines scored (n = 400) (Figure 4B). In addition, a minor fraction of germlines consisted of only mitotic germ cells (tumorous phenotype, data not shown). The extensive disorganization and decompaction of germline chromatin we observed in the majority of *top-2(it7);set-2* double mutants (Figure 4A,B) shows that chromatin architecture is significantly altered in the germline of these animals.

In wildtype oocytes, individual chromosomes appear as 6 condensed DAPI stained structures [28]. *top-2(it7)* mutants had a significant number of oocytes (67%) with more than 6 DAPI-stained bodies, and a smaller number (33%) with fewer (Figure 4C,D), consistent with the established role of topoisomerase II in sister chromatid cohesion [53]. A similar phenotype was observed in *top-2(it7);set-2* double mutants, suggesting that topoisomerase II acts independently of SET-2 in sister chromatid resolution. Altogether, our data suggest that SET-2 may contribute to the organization of pachytene chromosomes, together with condensin-II and Topo II.

### 2.5. Set-2 Inactivation Does Not Affect Germline Expression of Condensins, Topoisomerase, or Other Genes with a Known Role in Chromosome Structure or Segregation

To establish to what extent altered expression of condensins or other genes implicated in chromosome structure or segregation may contribute to the observed phenotypes of *set-2* mutant animals, we carried out RNA-sequencing (RNA-seq) on dissected gonads from *set-2/set-2* homozygotes derived from *set-2/+* heterozygous mothers. At a p value at ≤0.05 and ±0.58 log2FC (1.5 fold), 251 genes were found to be down-regulated, and 182 were up-regulated (Table S1). This relatively small number of genes is consistent with results from expression profiling of COMPASS mutants in other species [20,21,22,23]. Condensin subunits, topoisomerase, and other genes with known functions in chromosome structure or segregation were absent from our list of misregulated genes. These analyses suggest that the chromosome organization defects we observe in *set-2* mutant germlines are unlikely to simply reflect decreased expression of these genes, although we cannot formally exclude smaller, additive effects on the transcription of these or other genes in single or double mutants. In addition, because transcription has emerged as a major contributor to chromatin architecture [54,55], small alterations in gene expression patterns in *set-2* mutant germlines may also contribute to changes in chromatin architecture in these mutants.

### 2.6. Loss of COMPASS Targeting Subunit CFP-1 Results in Similar Chromatin Organization Defects as Loss of SET-2

**Figure 5:**
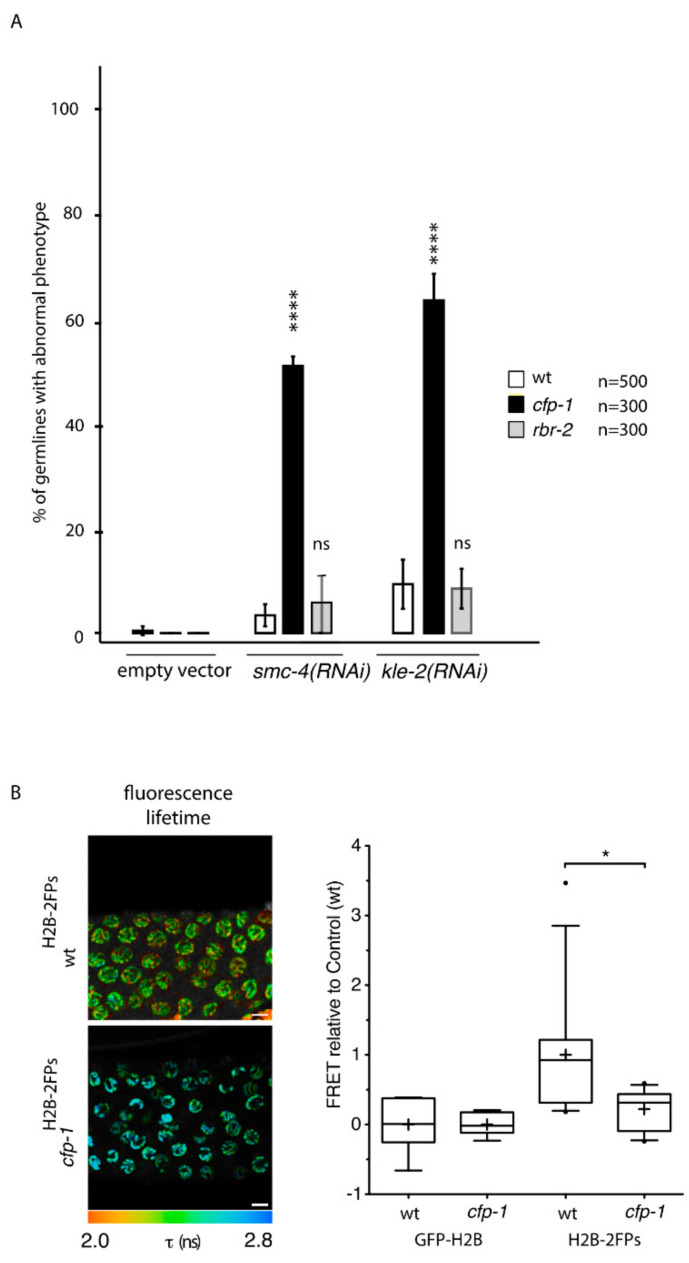
Inactivation of COMPASS targeting component CFP-1 mimics loss of SET-2. (**A**) Percentage of germlines with “strong” phenotype after RNAi directed against condensin-II (*smc-4* or *kle-2*) in wildtype, *cfp-1(tm6369)* and *rbr-2(tm1231)* mutants. n= number of animals scored from at least 3 independent experiments. [****] *p* < 0.0001 (*t*-test); ns, not significant. (**B**) Representative fluorescence lifetime images of wt ^H2B−2FPs^ and *cfp-1* ^H2B−2FPs^ mutant germlines and fluorescence lifetime values (τ). Scale bars, 10 μm. Box-and-whisker graph plots showing the decrease in FRET efficiency in *cfp-1* mutant animals. Mean FRET values ate indicated by a cross in each box. [*] *p* < 0.05 (two-tailed unpaired *t* test). n = 13 gonads for wt, n = 11 gonads for *cfp-1*.

To establish whether SET-2 contributes to germline chromatin organization in the context of COMPASS, we next asked whether other subunits of the complex also enhance the germline defects resulting from condensin-II knockdown. As observed for *set-2*, inactivation of *cfp-1* using either the knock-out allele *tm6369* or RNAi, resulted in a strong decrease in H3K4me3 in both the germline and soma [16,18,56,57] (Figure S7A). The number of animals with an abnormal germline phenotype following RNAi of *smc-4*, targeting condensin-I and -II, or *kle-2*, targeting condensin-II only, was largely increased in *cfp-1* mutants compared to wildtype (Figure 5A). FLIM-FRET analysis of *cfp-1* mutants expressing H2B-2FP showed a significant nanoscale decompaction of pachytene chromatin (Figure 5B), further supporting a role for COMPASS in chromosome organization.

H3K4me3 is removed by the well-conserved lysine demethylase RBR-2/KDM5 [58,59], which like *set-2* is required to maintain germline immortality at high temperatures [59]. We found that absence of RBR-2 activity in the *rbr-2(tm1231)* deletion allele [59] did not enhance germline defects resulting from either *kle-2* or *smc-4* (RNAi) (Figure 5A). qRT-PCR analysis confirmed that although the overall efficacy of RNAi varied between independent experiments, RNAi efficacy was comparable in wildtype, *cfp-1* and *rbr-2* mutants within the same experiment (Figure S7B). Furthermore, the percentage of animals with a strong phenotype was similar in all three experiments (Figure S7C), consistent with depletion of condensin-II below a threshold level being sufficient to provoke defects in chromosome organization [60]. Therefore, contrary to COMPASS inactivation, increasing H3K4 methylation levels in *rbr-2* mutants has no obvious impact on germline chromatin organization by this assay.

## 3. Discussion

Using two different experimental approaches, we found that in the *C. elegans* germline COMPASS contributes to the organization of chromosome architecture. First, using quantitative tagged-histone FLIM-FRET analysis we show that chromatin compaction at the nucleosomal level is strongly reduced in live animals that lack COMPASS subunits SET-2 or CFP-1. Second, we demonstrate that chromatin compaction defects in germline nuclei following knock-down of condensin-II or topoisomerase II are aggravated in the absence of COMPASS. Combined, these findings suggest a role for COMPASS in shaping chromosome architecture in *C. elegans.*

Although we cannot rule out that the defects in chromatin organization reflect a role for SET-2 in regulating the expression of relevant genes, our transcriptional profiling does not support such a model. Rather, we propose that COMPASS plays a more direct role in meiotic chromosome organization. We also consider a physical link between COMPASS and condensin unlikely, since we found no evidence for such an interaction in extensive proteomics analysis [19].

A current model proposes that condensin complexes topologically shape mitotic chromosomes through a loop extrusion process [61], with condensin-I and -II forming arrays of helical consecutive loops in mitotic cells [62]. However, the observation that chromosomes still maintain a certain degree of structure in the absence of both condensins suggests that additional mechanisms and factors may be involved, including histone modifying complexes and the associated modifications [60,63,64,65,66,67,68]. Using the same FLIM-FRET imaging approach implemented here, we previously showed that condensin complexes contribute to the nanoscale compaction of chromatin in the *C. elegans* germline [31]. Depletion of condensin-II only affected highly compacted regions, while depletion of condensin-I affected both highly- and lowly- compacted regions [31], similarly to loss of *set-2*. Because FRET in our system most likely results from clustering of distant regions from the same or different chromosomes, we propose that SET-2*,* and to a larger extent COMPASS, may contribute to the structural organization of meiotic chromosomes in a manner similar to Condensin-I. Because loss of the H3K9 methyltransferase MET-2 and the HP1 (Heterochromatin-Protein1) homolog HPL-2 resulted in a similar decrease in chromatin compaction measured by FLIM-FRET [31], some of the effects reported here may reflect altered H3K9 methylation patterns and heterochromatin distribution in the germline of *set-2* mutant animals [17]. Our previous observation that *set-2* inactivation does not enhance *hpl-2* germline defects also argues against loss of *set-2* generally enhancing germline defects associated with loss of chromatin associated factors [57].

In mammals, partial inactivation of condensin-II results in relatively moderate defects in chromosome structure, in contrast to the more severe defects resulting from its complete inactivation [40,64,69]. Using a similar approach, we found that the chromosome organization defects resulting from knockdown of condensin-II are significantly aggravated in the absence of *set-2* or *cfp-1*, consistent with COMPASS contributing to proper meiotic chromatin architecture together with condensin-II. We further confirmed that COMPASS and condensin-II functionally interact in the germline by showing that reduced fertility of the hypomorphic allele *hcp-6*(*mr17*) is aggravated in the absence of *set-2*. Interestingly, by resequencing the *hcp-6*(*mr17*) allele, we identified a mutation within one of the α-helical HEAT repeats of HCP-6, supporting their functional importance in condensin recruitment to chromatin [70,71,72].

We also found that *set-2* partially suppressed the embryonic lethality of *hcp-6*(*mr17*) mutants at the non-permissive temperature, an effect that may be due in part to the elimination of defective germ cells by increased apoptosis in *set-2;hcp-6* double mutant germlines. We note that mutations in the BRCA1 homologs *brc-1* or *brd-1* are also able to partially suppress the embryonic lethality of *hcp-6*(RNAi) animals, possibly reflecting a role for BRC-1/BRD-1 in the formation of toxic chromatin bridges when chromosome condensation is defective [73]. *set-2* may also be involved in a similar process in embryos.

Further insight into a likely role of SET-2 in chromatin architecture comes from the observation that its inactivation also enhanced the chromosome organization defects of *top-2* conditional mutants. In *C. elegans*, TOP-2 localization along mitotic chromosomes is thought to constrain chromosome length by modulating chromatin loops [74]. Topoisomerase II also localizes along the chromosome axes of meiosis I chromosomes, both in yeast and mammals [51,75,76]. Our results suggest that it may play a similar structural role in organizing *C. elegans* germline nuclei in cooperation with SET-2.

**Figure 6:**
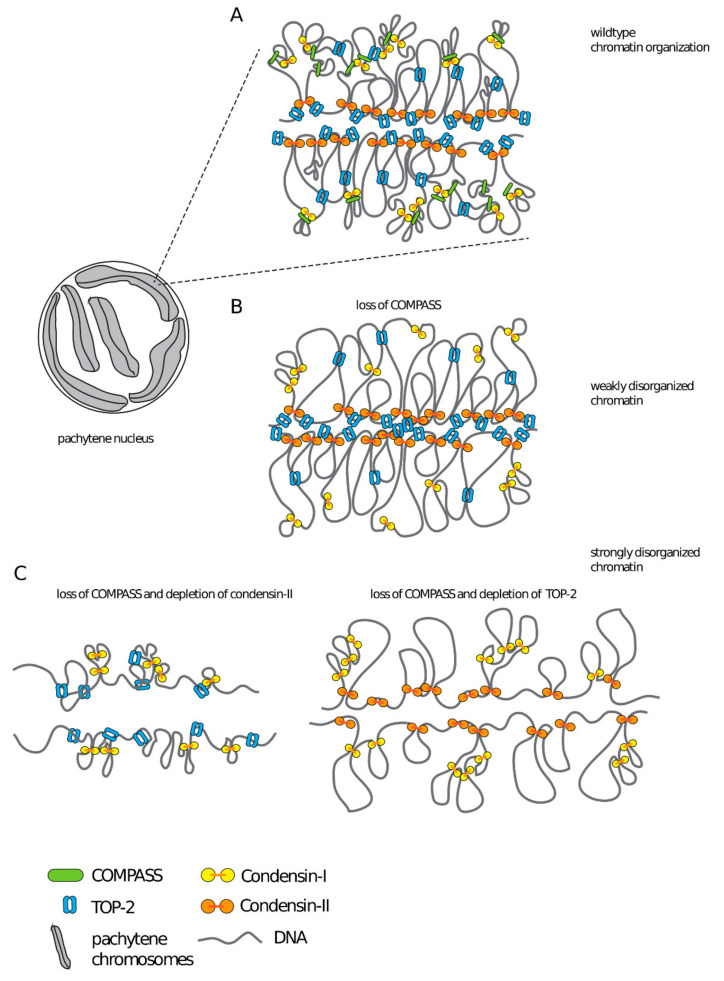
Working model for cooperation between COMPASS, condensin-II and Topoisomerase-II in chromosome organization. (**A**) In wildtype, proper chromosome compaction results from the activity of condensin-I (connected yellow circles), condensin-II (connected orange circles) and Topoisomerase-II (blue broken ring). The concerted action of condensins results in the formation of arrays of helical loops, with condensin-II generating outer loops and condensin-I forming inner loops [62]. Topoisomerase-II may contribute to compaction by modulating chromatin loops [74], or actively introducing self-entanglement in DNA [84]. COMPASS could mediate interactions between loops, possibly by contributing to the clustering of transcribed loci [82]. (**B**) Absence of COMPASS may result in subtle defects in chromosome organization (weakly disorganized chromatin), but the action of condensins, Topoisomerase-II and additional proteins would maintain overall chromosome architecture (**C**). We propose that partial depletion of condensin-II or Topoisomerase-II in the absence of COMPASS results in cumulative defects in chromosome organization. The model does not take into account heterogeneity between individual nuclei.

Based on our data, we propose that COMPASS contributes to the shaping of meiotic chromosomes, together with structural components condensin-II and topoisomerase II (Figure 6). Meiotic chromosomes are organized as linear loop arrays around a proteinaceous chromosome axis [77,78,79,80], with H3K4me3 emanating radially in similar structures that contribute to the shaping of meiotic chromosomes [81]. Furthermore, in mouse spermatocytes local interactions between linear loops are thought to reflect clustering of highly transcribed loci [82]. Based on these observations, we propose that the presence of COMPASS at transcription sites could potentially contribute to the organization of chromatin in these clusters, either through the recruitment of an H3K4me3 reader [83], through modifications in the chromatin landscape brought about by SET-2 dependent changes in gene expression [54,55], or through the recruitment of additional proteins [19]. Future experiments will investigate these non-mutually exclusive mechanisms.

## 4. Conclusions

Our studies highlight an important role for the evolutionarily conserved COMPASS complex in chromosome organization in the *C. elegans* germline. Deciphering the mechanism whereby chromatin-associated factors and histone post-translational modifications affect chromatin architecture in meiosis will be an important area of future study.

## 5. Methods

### 5.1. Nematode Maintenance and Strains

Unless otherwise noted, animals were propagated under standard conditions at 20 or 15 °C [85] on NGM plates (Nematode Growth Medium) seeded with the *Escherichia coli* strains OP50 or HT115 for RNAi experiments. N2 Bristol was used as the wildtype control strain. Strains used were as follows:

*hcp-6(mr17)* I (PFR656), *set-2(bn129)* III/qC1 (PFR510), *hcp-6(mr17)* I; *set-2(bn129)* III (PFR651), *cfp-1(tm6369)* IV (PFR588), *brc-1(tm1145)* III (DW102), *rbr-2(tm1231)* IV (PFR394), oxIs279[Ppie 1::GFP::H2B + *unc-119*(+)] II; *unc-119(ed3)* III (EG4601), oxI*s279*[P*pie-1*::GFP::H2B + *unc-119*(+)] II; *set-2(bn129)* III (PFR326), *oxIs279*[P*pie-1*::GFP::H2B + *unc-119*(+)] II; *cfp-1(tm6369)* IV (PFR667), oxSi487 [mex-5p::mCherry::H2B::tbb-2 3’UTR::*gpd-2* operon::GFP::H2B::*cye-1* 3’UTR + *unc-119*(+)] II; *unc-119(ed3)* III (EG6787), *oxSi487* [mex-5p::mCherry::H2B::tbb-2 3’UTR::*gpd-2* operon::GFP::H2B::cye-1 3’UTR + *unc-119*(+)] II; *set-2(bn129)* III (PFR659), oxSi487 [*mex-5*p::mCherry::H2B::*tbb-2* 3’UTR::*gpd-2* operon::GFP::H2B::*cye-1* 3’UTR + *unc-119*(+)] II; *cfp-1(tm6369)* IV (PFR666), *unc-119*(ed3) III; *top-2(it7)* II (PFR704), *top-2(it7)* II; *set-2(bn129)* III (PFR705)

### 5.2. Worm Preparation for Live-Imaging

For FRAP and FLIM-FRET acquisitions, single worms (24 h post-L4 stage) from an unsynchronized population were picked to an unseeded 1xNGM plate to wash off bacteria and were subsequently transferred onto a glass slide in a drop of egg buffer (118 mM NaCl, 48 mM KCl, 2 mM CaCl_2_^*^2H_2_O, 2 mM MgCl_2_^*^6H_2_O, 25 mM HEPES pH 7.3). Worm gonads were extruded by microdissection using a 23G syringe and immediately covered with a coverslip, sealed with nail varnish.

### 5.3. FLIM-FRET Data Acquisition

FLIM-FRET measurements were carried out on wt, *set-2(bn129) and cfp-1(tm6369)* mutant strains GFP-H2B (donor alone: GFP-H2B protein) and H2B-2FPs (donor and acceptor: GFP-H2B and mCherry-H2B). FLIM was performed using an inverted laser scanning multi-photon LSM780 microscope (Carl Zeiss Microscopy GmbH-Jena, Germany) equipped with an environmental black-walled chamber. Measurements were performed at 20 °C with a 40× oil immersion lens, NA 1.3 Plan-Apochromat objective, from Zeiss. Two-photon excitation was achieved using a tunable Chameleon Ultra II (680–1080 nm) laser (Coherent, Inc. Santa Clara, CA 95054, USA) to pump a mode-locked, frequency-doubled Ti:sapphire laser that provided sub-150-fs pulses at an 80-MHz repetition rate. GFP and mCherry fluorophores were used as a FRET pair. The optimal two-photon excitation wavelength to excite the donor GFP was determined to be 890 nm [30]. Laser power was adjusted to give a mean photon count rate of about 7 × 10^4^–10^5^ photons per second. Fluorescence lifetime measurements were acquired over 60 s. Detection of the emitted photons was achieved through the use of an HPM-100 module (Hamamatsu R10467-40 GaAsP hybrid photomultiplier tube [PMT]). And fluorescence lifetimes were calculated for all pixels in the field of view (256 × 256 pixels). The fluorescence lifetime imaging capability was provided by time-correlated single- photon counting (TCSPC) electronics (SPC-830; Becker & Hickl, Berlin, Germany). TCSPC measures the time elapsed between laser pulses and the fluorescence photons. Specific regions of interest (e.g., full gonad or pachytene nuclei) were selected using SPCImage software (Version 7.3; Becker & Hickl GmbH-Berlin, Germany). For the FRET analysis, we performed a manual segmentation by outlining pachytene nuclei to prevent any potential contribution of autofluorescence lifetime [86].

### 5.4. FLIM-FRET Analysis

FLIM measurements were analyzed as described previously [31] using SPCImage software (Becker & Hickl GmbH-Berlin, Germany). Briefly, FRET results from direct interactions between donor and acceptor molecules [87] and causes a decrease in the fluorescence lifetime of the donor molecules (GFP). The FRET efficiency (i.e., coupling efficiency) was calculated by comparing the fluorescence lifetime values from FLIM measurements obtained for GFP donor fluorophores in the presence and absence of mCherry acceptor fluorophores. The FRET percentage images were calculated such as, *E FRET* = 1 − (τ*DA*/τ*D)*, where τDA is the mean fluorescence lifetime of the donor (GFP-H2B) in the presence of the acceptor (mCherry-H2B) expressed in *C. elegans*^H2B−2FPs^, and τD is the mean fluorescence lifetime of the donor (GFP-H2B) expressed in *C. elegans*^GFP-H2B^ in the absence of the acceptor. In the non-FRET conditions, the mean fluorescence lifetime value of the donor was calculated from a mean of the τ_D_ by applying a mono-exponential decay model to fit the fluorescence lifetime decays.

### 5.5. Condensin RNAi Knockdown

Bacterial clones expressing RNA targeting condensin-I and -II subunit were from the *C. elegans* RNAi collection (Ahringer laboratory-Source BioScience, Nottingham, UK). Inserts from each RNAi clone were amplified by PCR on isolated colonies, with a single primer in the duplicated T7 promoter (5′ TAATACGACTCACTATAGGG 3′), then sequenced using the primer 5′ GGTCGACGGTATCGATAAGC 3′. RNAi clones were cultured in LB liquid medium supplemented with 50 µg/mL Ampicillin for 18 h at 37 °C, IPTG was then added (1mM final), and cultures grown an additional 2 h 30 min at 37 °C. NGM plates complemented with IPTG (1 mM) were seeded with 300 μL of bacterial culture. Synchronized L1 were placed on RNAi plates and grown to adulthood.

### 5.6. RNA Isolation and qRT-PCR Analysis

Synchronized L1 wildtype or *set-2(bn129)* mutant worms were grown on empty vector L4440 or *kle-2* or *capg-1* RNAi to adult staged worms at 20 °C and harvested. Total RNA was isolated using NucleoZol (Macherey Nagel GmbH, Düren, Germany; #740404-200) and NucleoSpin (Macherey Nagel GmbH, Düren, Germany; #40609). RNA was reverse transcribed using cDNA transcriptor (Roche, #5893151001). Quantitative PCR analysis was performed on CFX Connect (Bio-rad, Hercules, CA 94547, USA) with SYBR Green RT-PCR (Roche, Basel, Switzerland; #4913914001). Melting curve analysis was performed for each primer set to ensure the specificity of the amplified product and with an efficiency of 2. *pmp-3* and *cdc-42* were used as the internal controls so that the RNA level of each gene of interest was normalized to the levels of *pmp-3* and *cdc-42*. qRT-PCR were performed on three biological replicas in technical duplicates. Statistical analysis was performed using an unpaired *t*-test. Primers used were:

*pmp-3*: 5′ GTTCCCGTGTTCATCACTCAT 3′-5′ ACACCGTCGAGAAGCTGTAGA 3′

*cdc-42*: 5′ CTGCTGGACAGGAAGATTACG 3′-5′ CTCGGACATTCTCGAATGAAG 3′

*kle-2:* 5′ GAGAAAACGGACAGCTCGTGTG 3′-5′ CGTCATATTCAGCTCCGAGGGT 3′

*capg-1:* 5′ TCGAATTGGCCAGTAGATGC 3′-5′ ACTGCAACAAGTCGGCATTC 3′

### 5.7. Hoechst Staining on Dissected Germlines

Germlines from condensin RNAi knock-down animals were dissected on L-polylysine coated slides in a drop of dissection buffer (0.4× M9 and Levamisole 20 mM). After removing dissection buffer using a drawn capillary, gonads were fixed in 11 μL of 3% paraformaldehyde for 5min. Slides were washed in 1× PBS 0.2% Tween 20 plus 5 μg/mL Hoechst 33342 (Sigma Aldrich, #861405, St. Quentin Fallavier, France) for 10 min, then twice in 1× PBS 0.2% Tween 20 for 10 min, and mounted in mounting media (1× PBS, 4% n-Propyl-Gallate, 90% DE Glycerol). Z-stack images (0.25 μm slices) of germlines were acquired using a Zeiss LSM710 inverted confocal microscope with a 40× oil immersion objective.

### 5.8. DAPI Staining on Whole Animals

For scoring topo II mutant germlines, adult animals were stained as previously described [88] with minor modifications. Briefly, animals were collected and washed once in 1× M9, fixed 15 min in −20 °C methanol, and washed twice in 1× PBS with 0.1% Tween 20^®^ (Sigma Aldrich, #P1379, St. Quentin Fallavier, France). 25 μL Fluoroshield plus DAPI (Sigma Aldrich, #F6057, St. Quentin Fallavier, France) was added directly to 50 mL of worm pellet, followed by mounting for fluorescent microscopy. Observation were made on an AxioImager A2 (Carl Zeiss Microscopy GmbH-Jena, Germany) with Plan Apochromat 63×/1.4 oil DIC or EC plan Neofluar 20×/0.5 objectives.

### 5.9. Scoring of Germline Phenotypes

Blind scoring was carried out using AxioImager A2 (Carl Zeiss Microscopy GmbH-Jena, Germany) with EC plan Neofluar 20×/0.5 objectives. For condensin RNAi knock-down, the “abnormal phenotype” was defined as germlines containing fewer, abnormally sized and unevenly distributed nuclei, as well as macro nuclei with strong DAPI signal, as previously described [39]. “wildtype-like” includes germlines that mostly resembled wildtype and sometimes contained a few macro nuclei with strong DAPI signal. For each experiment, at least 200 germlines were scored for each genotype, and at least 3 independent biological replicates were performed. For experiments with the *top-2(it7)* allele, wildtype, *set-2(bn129)* and *top-2(it7)* single, and *set-2(bn129);top-2(it7)* double mutants were synchronized at the L1 stage at 15 °C, then transfer on plates seeded with OP50 at 24 °C and allowed to develop to adulthood. Adults were recovered in 1× M9 and DAPI stained as described in DAPI staining on whole animals. Germlines were place in phenotypic categories based on [89]. Data were collected from 3 independent experiments.

### 5.10. Sequencing and Mapping of the Hcp-6(mr17) Mutation

Genomic DNA from wildtype animals (N2) and from strains bearing the *hcp-6(mr17)* mutation was amplified using a high-fidelity polymerase (Phusion^®^, NEB #M0530S, Ipswich, MA, USA) and the following primer pairs:

5′ ATAGTCAACCTCGATTGCTGGCTG 3′-5′ GAGGGCGAATAAGTCTTCCGTAAG 3′

5′ GGAGTTTCTGCTGCCAGTAGTTAT 3′-5′ TGTGGATAAACGTGGCGATA 3′

5′ GATCGTTGGAGCGATTTACGGATC 3′-5′ TGTGGATAAACGTGGCGATA 3′

5′ GATCGTTGGAGCGATTTACGGATC 3′-5′ CTTTCTGGCATGTTCAGTGACGTC 3′

5′ GAAATCCCGAAGCAAGAGAG 3′-5′ GTCCATGTGAGATCCGATGAGT 3′

5′ GAAATCCCGAAGCAAGAGAG 3′-5′ CTTTCTGGCATGTTCAGTGACGTC 3′

5′ TGGCTTCACACCTTGATCTCGATG 3′-5′ TCTTCATCGTGACCAACTCCAACC 3′

5′ TCTCAACGTGGCATCTGAAG 3′-5′ GCGTGTCGACGAACAATAAC 3′

5′ GTTCGGAATGACGCAAAACT 3′-5′ CACAGTTTTCTCCGCATCAACATG 3′

5′ CACTGAAATGCGCCTTAATCCTCC 3′-5′ TGATATGGGAGGAGCTGTGAAGGA 3′

For each DNA fragment amplified by PCR, both forward and reverse primers were used in the sequencing reactions. The presence of the *mr17* mutation was confirmed in 7 independent sequencing reactions from independent isolates. 2 independent reactions from wildtype animals were used as reference.

### 5.11. Brood Size and Embryonic Lethality Assays

To score fertility and embryonic lethality, 10 to 11 individual L4 hermaphrodites grown at 15 °C were picked and transferred to individual plates at either 15, 20 or 25 °C. Animals were transferred on new plates until they stopped laying eggs, and the number of eggs on individual plates scored each day. After 24 h, unhatched eggs and live progeny were scored. Embryonic lethality represents the number of unhatched eggs, divided by the total number of total eggs laid. Experiments were repeated 3 times each.

### 5.12. Visualization of Apoptotic Cells in the Germline

Acridine Orange (Sigma Aldrich #A9231, St. Quentin Fallavier, France) was used to visualized apoptotic cells in the germline of live animals as previously described [90]. Briefly, L4 hermaphrodites grown at permissive temperature (15 °C), were placed on NGM plates at the restrictive temperature of 20 °C during 18 h. 1ml of Acridine Orange diluted at a final concentration of 50 μg/mL in M9 buffer was added to the plates and incubated for 2 h in the dark. Stained animals were transferred to a fresh NGM plates seeded with OP50 and incubated for 2 h in the dark in order to remove stained bacteria in the intestine. Animals were placed on 4% agar pad in a drop of 10 mM levamisole (Sigma Aldrich #L9756, St. Quentin Fallavier, France) diluted in M9 buffer, a coverslip was placed on top and sealed with nailed polish. Z-stack images of the posterior gonad were acquired using a Zeiss LSM710 inverted confocal microscope with 40× oil Immersion objective. Z-stack of germlines were acquire every 0.5 μm, images correspond to a projection using Max intensity method using Fiji [91]. At least 20 gonads were imaged for each genotype.

### 5.13. RNA Sequencing of Dissected Gonads

Gonad dissections and extractions were performed as in [17]. Briefly, prior to dissection worms were placed on NGM plates without food to expel bacteria from the gut. Gonads of 5 to 7 young adults at the L4 stage + 12 h were dissected in dissection buffer (Egg Buffer1.1 × (HEPES pH 7.3 25 mM, NaCl 118 mM, KCL 48 mM, CaCl_2_ 2 mM, 2 mM MgCl_2_), 0.5 mM Levamisole, 0.1% Tween 20) on slides. Extruded gonads were cut at the elbow and the distal part recovered using a drawn capillary and transferred to 30 µL of ×B extraction buffer (Kit Picopure, Life Technology, # 12204-01, Carlsbad, MA, USA), frozen in liquid nitrogen and stored at −80 °C. For RNA preparation tubes were thawed, the volume of XB extraction buffer adjusted to 100 μL, and RNA purified using the PicoPure kit (Life Technology, # 12204-01) according to the manufacturer’s instructions. Elution was in 13 μL of nuclease-free water. The integrity of RNA was evaluated using Tape Station 4200 (Agilent, Santa Clara, CA, USA), and the concentration of RNA measured using DropSense 96 (Unchained Labs, Pleasanton, CA, USA). Construction of rRNA depleted libraries was carried out at the GenomEast platform (IGBMC, Strasbourg, France), and sequencing by an Illumina Hiseq 4000 device.

Bioinformatic analysis was carried out under Galaxy [92]. Sequence reads were mapped onto the reference genome (WS254) with the RNA-STAR tool (Version 2.4.1d). Sequences with a quality of cartography lower than 10 were removed with SAMtools (Version0.1.19). The expression level of each gene for each sample was calculated with htseq-count (Version 0.7.2). Differential analysis of gene expression between the different strains was carried out with the DESeq2 package version 1.16.1 [93] under R version 3.4.4. Additional analyzes were performed with R. Data may be viewed at https://www.ncbi.nlm.nih.gov/geo/query/acc.cgi?acc=GSE146932.

## Supplementary Materials

The following are available online at https://www.mdpi.com/2073-4409/9/9/2049/s1, Figure S1: *set-2* inactivation differentially impacts H3K4me3 in the germline, Figure S2: Fluorophore tagged histones H2B are correctly expressed and incorporated into chromatin in *set-2* mutants, Figure S3: Polyploid cells resulting from *kle-2* knockdown in *set-2* mutant germlines, Figure S4: Condensin-I depletion in wildtype and *set-2* mutant animals results in similar reduction in body size, Figure S5: Germline defects of *hcp-6(mr17)* mutant animals and suppression of *hcp-6(mr17)* embryonic lethality, Figure S6: Distribution of *set-2* misregulated genes in different expression classes, Figure S7: Loss of H3K4me3 in *cfp-1* mutant germline and efficacy of *cfp-1* and *rbr-2* RNAi in independent experiments. Table S1: Transcriptional profiling (RNA-seq) of *set-2* mutant germlines.
